# An Unusual Complication at an Unusual Site: Internal Jugular Vein Thrombosis Revealing Celiac Disease

**DOI:** 10.7759/cureus.101161

**Published:** 2026-01-09

**Authors:** Meriem Mouharir, Zakaria Chahbi, Hassan Qacif, Said Kaddouri, Mohamed Zyani

**Affiliations:** 1 Department of Internal Medicine, Avicenne Military Hospital - Cadi Ayyad University, Marrakesh, MAR; 2 Department of Internal Medicine, Ibn Sina Military Hospital, Marrakesh, MAR

**Keywords:** celiac disease, hyperhomocysteinemia, internal jugular vein thrombosis, thrombosis, unusual site

## Abstract

Celiac disease is now recognized as a common disorder that can be diagnosed at any age and affects many organ systems. Its clinical expressions can appear as digestive or extra-digestive; among these, thromboembolic events are the focus of our study. We report a case of a 63-year-old woman who presented with left-sided cervical swelling associated with edema of the left upper limb, with generalized weakness and weight loss. The clinical examination revealed a tender cervical mass in the region of the internal jugular vein. Doppler ultrasound demonstrated thrombosis of the left internal jugular vein extending into the subclavian vein, in the absence of classical risk factors for venous thrombosis. Laboratory investigations showed iron deficiency microcytic anemia and a biological malabsorption profile. Celiac disease was suspected and confirmed by positive serology and duodenal biopsies showing villous atrophy (Marsh 3C). The patient was treated with anticoagulation and a strict gluten-free diet, with a favorable clinical evolution. This case highlights celiac disease as a potential underlying cause of unexplained unusual-site venous thrombosis.

## Introduction

Celiac disease (CD) is a chronic, immune-mediated enteropathy triggered by gluten in genetically predisposed individuals. It can present with a broad spectrum of intestinal manifestations (chronic diarrhea, weight loss, and malabsorption syndrome) and extra-intestinal complications, including oral aphthous stomatitis, articular symptoms, amenorrhea, and thromboembolic events [1-3]. Although an increased risk of venous thromboembolism has been reported in patients with CD, venous thrombosis rarely represents the initial presentation, especially when it occurs at an unusual site [3,4]. We report a case of internal jugular vein thrombosis revealing previously undiagnosed CD in an adult woman.

## Case presentation

We present a case of a 63-year-old woman with a medical history of unexplored iron deficiency anemia. She was admitted for left-sided cervical swelling associated with edema of the left upper limb. She was afebrile with generalized weakness and weight loss. She had no identifiable thrombotic risk factors, including no history of recent surgery, no immobilization, no trauma, no central venous catheterization, no use of estrogen-progestin contraceptives, no smoking, or known malignancy. On physical examination, she presented with mucocutaneous pallor and a non-pulsatile, tender left cervical swelling, associated with edema of the ipsilateral upper limb. The vital signs were stable, she was afebrile, and her body mass index was 17 kg/m².

Doppler ultrasound demonstrated thrombosis of the left internal jugular vein extending into the ipsilateral subclavian vein. There were no signs of local neck infection or mass. Laboratory investigations showed iron deficiency microcytic anemia with hemoglobin at 9 g/dL and ferritin at 10 ng/mL, as well as lymphopenia at 800/mm³. The remainder of the malabsorption workup revealed hypocholesterolemia, hypoproteinemia, hypoalbuminemia, hypocalcemia, and vitamin B12 and folate deficiency (Table 1). Renal and liver function tests were normal.

**Table 1:** Laboratory findings of the patient.

Laboratory tests	Patient laboratory values	Reference values
Hemoglobin (g/dL)	9.1	13.0-17.0
Mean corpuscular volume (%)	61	80.0-98.0
Mean corpuscular hemoglobin (pg)	23	26.0-34.0
Neutrophil polynuclear/mm^3^	5160	1400-7700
Lymphocyte/mm^3^	800	1000-4800
Platelets/mm^3^	249000	150000-450000
Hypocalcemia (mmol/L)	1.58	2.12-2.55
Hypoalbuminemia (g/L)	27	35-50
Hypoproteinemia (g/L)	57	62-87
Ferritinemia (ng/mL)	10	30-400
Hypocholesterolemia (g/L)	1.18	1.4-2.0
Vitamin B12 (pg/mL)	<141	165-489
Folates (ng/mL)	2.36	4.8-17.0
Anti-transglutaminase IgA antibody (U/mL)	245	<4
Anti-endomysial IgG antibody	Positive	<1/40
Protein C (%)	72	70-140
Protein S (%)	83	70-130
Anti-cardiolipin antibodies IgM (U/mL)	0	<10
Anti-cardiolipin antibodies IgG (U/mL)	0	<10
β2-glycoprotein I antibodies IgM (U/mL)	0	<10
β2-glycoprotein I antibodies IgG (U/mL)	6	<10
Lupus anti-coagulant	Negative	-
Homocysteine (μmol/L)	60	6-17
Anti-nuclear antibodies	Negative	<80

As part of the etiological assessment of this unusual-site venous thrombosis, breast ultrasound and mammography were normal. Screening for inherited and acquired thrombophilia did not reveal any abnormality, except for elevated plasma homocysteine levels consistent with hyperhomocysteinemia (Table 1). An autoimmune workup for anti-phospholipid syndrome, including lupus anticoagulant and anti-cardiolipin/β2-glycoprotein I antibodies, was negative.

**Figure 1:**
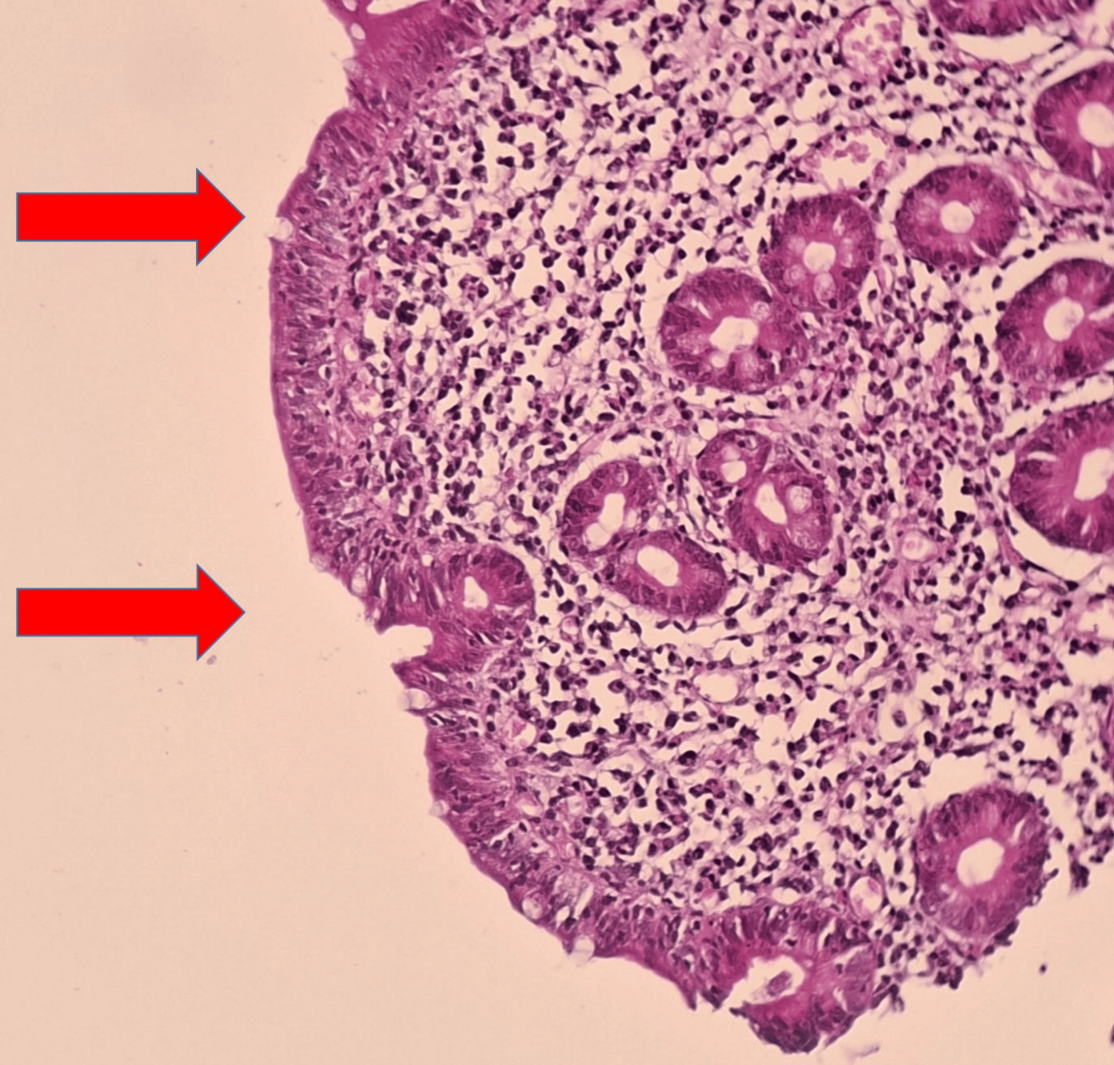
Light microscopy (×100) of duodenal mucosa showing chronic duodenitis with total villous atrophy, crypt hyperplasia, and marked intraepithelial lymphocytosis (arrows).

**Figure 2:**
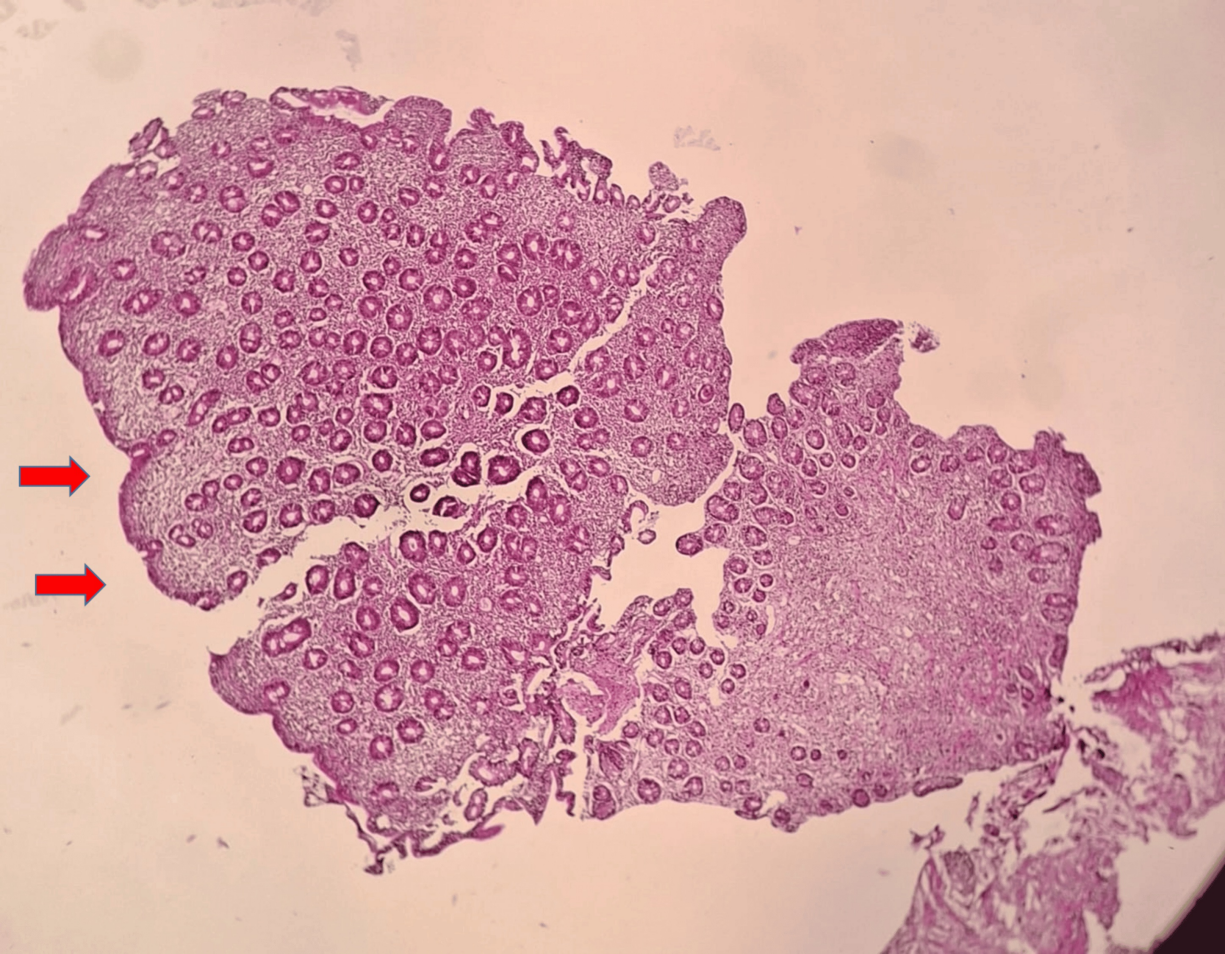
Light microscopy (×40) of duodenal mucosa showing chronic duodenitis with total villous atrophy, crypt hyperplasia, and marked intraepithelial lymphocytosis (arrows).

In view of the long-standing refractory iron deficiency anemia and biological evidence of malabsorption, celiac disease was suspected. Immunological analysis found positive anti-transglutaminase IgA and anti-endomysial IgG antibodies. Upper gastrointestinal endoscopy showed flattened duodenal folds. Histological examination of duodenal biopsies revealed total villous atrophy and an increased number of intra-epithelial lymphocytes (35%), consistent with celiac disease (Figures 1, 2).

The diagnosis of celiac disease, revealed by internal jugular vein thrombosis, was established. The patient was therefore started on anticoagulant therapy with a direct oral anticoagulant (rivaroxaban), with injectable iron and a strict gluten-free diet. She was monitored regularly. Clinical evolution was favorable, with regression of cervical swelling and improvement in general condition. Follow-up laboratory tests showed progressive correction of anemia and improvement of nutritional markers.

## Discussion

Internal jugular vein thrombosis (IJVT) is an uncommon but potentially serious manifestation of venous thromboembolism and is most frequently associated with central venous catheterization, head and neck infections (such as Lemierre’s syndrome), trauma, or malignancy [2]. In the absence of these classical local or systemic risk factors, IJVT should prompt a systematic search for underlying prothrombotic conditions, including inherited and acquired thrombophilias, systemic inflammatory diseases, and occult cancer [1,2]. In our patient, none of the usual predisposing factors were identified, and the etiological evaluation ultimately led to the diagnosis of previously unrecognized celiac disease (CD).

CD is now recognized as a common immune-mediated enteropathy that can be diagnosed at any age and may involve multiple organ systems. Beyond its classical digestive presentation, it is increasingly reported to be associated with venous thromboembolism [3]. The association between CD and thrombotic events has been increasingly recognized over the last two decades [3]. More than half of events occur without obvious provoking factors. Thrombosis frequently arises at atypical sites, particularly in the splanchnic circulation and hepatic veins, and may precede the diagnosis of CD in a substantial proportion of patients [3-6]. Venous thromboembolic events appear to be more common than arterial thrombosis in CD; hepatic vein thrombosis (Budd-Chiari syndrome) is among the most frequently reported sites, followed by lower-limb deep vein thrombosis, pulmonary embolism, and cerebral venous thrombosis [3,7,8].

The prothrombotic state in CD is multifactorial [3,9]. Several mechanisms have been proposed as follows: nutritional deficiencies (vitamin K-dependent protein C and protein S deficiency; folate and vitamin B12 deficiency leading to hyperhomocysteinemia), genetic predisposition, such as MTHFR variants, thrombophilic autoantibodies (anti-cardiolipin, anti-β2-glycoprotein I, anti-prothrombin and anti-phosphatidylserine/prothrombin), endothelial dysfunction, and platelet abnormalities [1,4,9,10]. Most patients had at least one acquired prothrombotic abnormality related to malabsorption, including hyperhomocysteinemia and protein C/S deficiency due to vitamin K deficiency, or anti-phospholipid antibodies [4,11].

Hyperhomocysteinemia deserves particular attention. It is a well-recognized prothrombotic factor associated with platelet activation, oxidative stress, endothelial dysfunction, and reduced levels of natural anticoagulants. In CD, hyperhomocysteinemia is typically secondary to malabsorption of folate and vitamin B12, and its prevalence at diagnosis has been reported to be around 20-30% in some adult series [9,10]. In our patient, acquired hyperhomocysteinemia, secondary to vitamin B12 and folate deficiency related to CD-associated malabsorption, was considered a major prothrombotic driver likely contributing to the internal jugular vein thrombosis.

Our observation is noteworthy for the unusual site of thrombosis (internal jugular veins) and for the fact that the thrombotic event revealed previously unrecognized adult CD. Most thrombotic complications reported in CD involve the splanchnic circulation (hepatic, portal, splenomesenteric veins) or cerebral thrombophlebitis; internal jugular vein thrombosis has been much less frequently described [5,7]. In addition, extensive evaluation of our patient did not identify inherited thrombophilia or anti-phospholipid syndrome, making the acquired abnormalities related to malabsorption (iron deficiency anemia, global protein and lipid depletion, hypocalcemia, hyperhomocysteinemia) the most plausible explanation for the prothrombotic state.

Clinicians should maintain a high index of suspicion for CD in patients with unexplained venous thromboembolism, especially at atypical sites, who present with chronic anemia, weight loss, or biological signs of malabsorption, even in the absence of overt gastrointestinal symptoms. Once CD is diagnosed, a systematic search for correctable prothrombotic factors (vitamin K, folate, and vitamin B12 deficiency, hyperhomocysteinemia, protein C/S deficiency, and anti-phospholipid antibodies) is warranted [4,12].

Management of thrombosis in CD relies on standard anti-coagulation combined with strict adherence to a gluten-free diet, which usually leads to mucosal healing, correction of malabsorptive deficiencies, and potentially, reduction of thrombotic risk over time [8,13]. The optimal duration of anti-coagulation in this setting remains uncertain and should be individualized, taking into account the presence of persistent thrombophilic factors, the severity of CD, and the site and extent of thrombosis. Decisions must be made on a case-by-case basis [4,8,13].

In summary, our observation illustrates that CD can present with internal jugular vein thrombosis, adding to the growing spectrum of atypical thrombotic manifestations of this disease. In patients with unexplained venous thrombosis, particularly at unusual sites, screening for CD and its associated acquired thrombophilic abnormalities should be systematically considered. Early diagnosis, institution of a gluten-free diet, correction of nutritional deficiencies, and appropriate anticoagulant therapy are essential to prevent recurrence and long-term complications.

## Conclusions

The diagnosis of CD should be considered in cases of unexplained thrombotic manifestations, even in the absence of digestive symptoms. In CD, the risk of thrombotic events can be explained by hyperhomocysteinemia, vitamin B12 and folate deficiency, genetic predisposition (such as MTHFR mutations), and acquired deficiencies of natural anticoagulants, including protein C and protein S. These risk factors may be acquired in the course of the disease and should be systematically investigated and corrected. A gluten-free diet remains the cornerstone of treatment for CD, and correction of nutritional deficiencies through vitamin supplementation, as well as thromboembolic prophylaxis, should be considered on a case-by-case basis.

## References

[1] Ramanandafy H, Ratsimbazafy SJ, Randrianarivony MH (2022). Prevalence and etiological profiles of atypical localization venous thrombosis: a descriptive multicenter study. [Article in French]. Ann Cardiol Angeiol (Paris).

[2] Salah RB, Frikha F, Kaddour N (2014). Risk factor for deep venous thrombosis in internal medicine: a retrospective study of 318 cases. [Article in French]. Ann Cardiol Angeiol (Paris).

[3] Chakor F, Achdami F, Handa A (2025). Thromboembolic complications unveiling celiac disease: a report of two cases. Sch J Med Case Rep.

[4] Berthoux E, Fabien N, Chayvialle JA, Ninet J, Durieu I (2011). Adult celiac disease with thrombosis: a case series of seven patients. Role of thrombophilic factors. [Article in French]. Rev Med Interne.

[5] Afredj N, Metatla S, Faraoun SA (2010). Association of Budd-Chiari syndrome and celiac disease. [Article in French]. Gastroenterol Clin Biol.

[6] Hriz FB, Habbassi H, Maamouri N (2010). Budd-Chiari syndrome associated with celiac disease. [Article in French]. Rev Med Interne.

[7] Zouiter S, Bensabbahia D, Atrassi M, Abkari A (2024). Cerebral thrombophlebitis complicating coeliac disease. Cureus.

[8] Pantic N, Pantic I, Jevtic D (2022). Celiac disease and thrombotic events: systematic review of published cases. Nutrients.

[9] Lerner A, Blank M (2014). Hypercoagulability in celiac disease - an update. Autoimmun Rev.

[10] Ferretti A, Parisi P, Villa MP (2013). The role of hyperhomocysteinemia in neurological features associated with coeliac disease. Med Hypotheses.

[11] Bahloul M, Chaari A, Khlaf-Bouaziz N (2005). Celiac disease, cerebral venous thrombosis and protein S deficiency, a fortuitous association?. [Article in French]. J Mal Vasc.

[12] Boucelma M, Saadi M, Boukrara H, Bensalah D, Hakem D, Berrah A (2013). Association of celiac disease and cerebral venous thrombosis: report of two cases. [Article in French]. J Mal Vasc.

[13] Abboud Y, Shah VP, Jiang Y, Pendyala N, Hajifathalian K (2024). Celiac disease is associated with increased risk of deep vein thrombosis and hypotensive shock in patients admitted with acute pancreatitis. JGH Open.

